# Induction of autophagy contributes to cisplatin resistance in human ovarian cancer cells

**DOI:** 10.3892/mmr.2014.2671

**Published:** 2014-10-16

**Authors:** LINGJIE BAO, MELBA C. JARAMILLO, ZHENBO ZHANG, YUNXI ZHENG, MING YAO, DONNA D. ZHANG, XIAOFANG YI

**Affiliations:** 1 Department of Gynecology, Obstetrics and Gynecology Hospital, Fudan University, Shanghai 200011, P.R. China; 2 Department of Obstetrics and Gynecology of Shanghai Medical School, Fudan University, Shanghai 200011, P.R. China; 3 Shanghai Key Laboratory of Female Reproductive Endocrine Related Diseases, Shanghai 200011, P.R. China; 4 Department of Pharmacology and Toxicology, University of Arizona, Tucson, AZ 85721, USA; 5 Department of Obstetrics and Gynecology, Shanghai First People’s Hospital, Jiaotong University, Shanghai 200080, P.R. China; 6 State Key Laboratory of Oncogenes and Related Genes, Shanghai Cancer Institute, Renji Hospital, Shanghai Jiao Tong University School of Medicine, Shanghai 200032, P.R. China

**Keywords:** ovarian carcinoma, cisplatin, autophagy, resistance

## Abstract

Cisplatin resistance is a major challenge in the clinical treatment of ovarian cancer, of which the underlying mechanisms remain unknown. The aim of the present study was to explore the role of autophagy in cisplatin resistance in ovarian cancer cells. A2780cp cisplatin-resistant ovarian carcinoma cells and the A2780 parental cell line, were used as a model throughout the present study. The cell viability was determined using a water soluble tetrazolium salt-8 assay, and western blot analysis was performed to determine the protein expression levels of microtubule-associated protein 1 light chain 3 (LC3 I and LC3 II), and Beclin 1. Beclin 1 small interfering (si)RNA and 3-methyladenine (3-MA) were used to determine whether inhibition of autophagy may re-sensitize cisplatin-resistant cells to cisplatin. The ultrastructural analysis of autophagosomes was performed using transmission electron microscopy, and apoptosis was measured by flow cytometry. In both A2780cp and A2780 cells, cisplatin induced the formation of autophagosomes and upregulated the expression levels of autophagy protein markers, LC3 II and Beclin 1. However, the levels of autophagy were significantly higher in A2780cp cells, as compared with the A2780 cells. The combined treatment of cisplatin with 3-MA, the autophagy pharmacological inhibitor, increased the cell death rate, but had no effects on apoptosis, as compared with cisplatin treatment alone in A2780cp cells. However, inhibition of autophagy by siRNA knockdown of Beclin 1 expression enhanced cisplatin-induced cell death and apoptosis. The findings of the present study suggest that autophagy has a protective role in human ovarian cancer cells, and that targeting autophagy may promote chemotherapeutic sensitivity.

## Introduction

Ovarian cancer is one of the most common gynecological malignancies. Currently, debulking surgery and cisplatin-based chemotherapy are the recommended approaches with the highest curative potential (1). However, acquired chemoresistance is a major obstacle that affects the success rate of ovarian cancer treatment. The five-year survival rate for ovarian cancer patients is currently ~30% (2). Previous studies have researched the molecular alterations in cisplatin-resistant cancer cells, however, the underlying mechanisms promoting cisplatin resistance in ovarian cancer cells remain to be elucidated (3).

Previous studies have suggested that autophagy may have a role in cancer cell chemoresistance (4,5). Autophagy is a cellular process of self destruction which occurs in all eukaryotic cells. Damaged organelles and molecules are engulfed by autophagosomes and degraded by lysosomal hydrolases, for energy recycling (6). Dysregulation of autophagy has been associated with numerous diseases, including cancer (7); however, the role of autophagy in cancer chemoresistance remains unknown.

Inhibition of autophagy by 3-methyladenine (3-MA), or Beclin 1 small interfering (si)RNA, was previously shown to increase chemotherapy-induced apoptosis in human hepatocellular carcinoma cells, and inhibit tumor growth in a mouse xenograft model (8). Furthermore, knockdown of Beclin 1 and autophagy-related protein 7 (ATG7) expression levels, in OE19 and KYSE450 esophageal cancer cells, enhanced the effects of 5-fluorouracil (5-FU), a chemotherapeutic agent used to treat esophageal cancer (9). These observations suggest that inhibitors of autophagy may be potential targets to improve the therapeutic efficacy of conventional chemotherapeutics. Conversely, in MCF-7 breast cancer cells, knockdown of migration inhibitory factor expression, was shown to enhance the cytotoxicity of doxorubicin and etoposide, by inducing autophagy (10). A previous study using H460/cis cisplatin-resistant lung carcinoma cells additionally showed that the levels of microtubule-associated protein 1 light chain 3 (LC3), and autophagosome formation were significantly lower in resistant cells, as compared with their non-resistant parental cells (11). Furthermore, a co-treatment of cisplatin with trifluoperazine, an inducer of autophagy, sensitized H460/cis cells to cisplatin, suggesting that the decreased levels of autophagy may promote cisplatin resistance in lung cancer. Currently, no consistent conclusions have been made regarding the role of autophagy in chemoresistance. Therefore, the aim of the present study was to investigate the role of autophagy in mediating cisplatin resistance in human ovarian cancer cells, and explore autophagy as a potential target for ovarian cancer treatment.

## Materials and methods

### Cell lines and reagents

The A2780 cisplatin-sensitive human ovarian cancer cell line, and the A2780cp cisplatin-resistant clone, were obtained from the Shanghai Key Laboratory of Female Reproductive Endocrine Related Diseases (Shanghai, China). The A2780 cisplatin-sensitive cells were cultured in Dulbecco’s modified Eagle’s medium (DMEM; Gibco Life Technologies, Carlsbad, CA, USA), supplemented with 10% fetal bovine serum (FBS South American origin; Bio-west, Logan, UT, USA), 100 U/ml penicillin and 100 μg/ml streptomycin (Sigma-Aldrich, St.Louis, MO, USA), in a humidified 5% CO_2_ atmosphere, at 37°C. A2780cp cells were grown in DMEM, supplemented with 10% FBS and 1 μg/ml cisplatin, to maintain resistance. The cisplatin was purchased from Hansoh Pharmaceutical Co., Ltd. (Lianyungang, Jiangsu, China), and the 3-MA (M9281; Sigma-Aldrich) was dissolved in sterile double distilled water at 65°C.

### Cell viability assay

The A2780 and A2780cp cells were seeded at 1×10^4^ cells/well, in 96-well plates. The following day, various concentrations (5–50 μg/ml) of cisplatin were added to the wells and the plates were incubated for 24 h. Each treatment was applied to four wells. The cell viability was assessed using a water soluble tetrazolium salt-8 (WST-8) Cell Counting kit (Dojindo Molecular Technologies, Inc., Kumamoto, Japan). Briefly, 10 μl WST-8 and 100 μl DMEM was added to each well and incubated for 2 h. The absorbance was measured at a wavelength of 450 nm, using a microplate reader (Model 680, Bio-Rad Laboratories, Hercules, CA, USA). Each experiment was repeated three times.

### Western blot analysis

Cells lysis, protein extraction and quantification were performed as previously described (12). Equal amounts of protein (20 μg), from the harvested cells, was loaded onto 10–15% w/v polyacrylamide gels and separated by SDS-PAGE, followed by transfer to polyvinylidene fluoride membranes (Millipore, Billerica, MA, USA). The membranes were blocked with 5% non-fat milk in phosphate-buffered saline (PBS), at room temperature for 1 h. Subsequently, the membranes were incubated overnight at 4°C, with a 1:1,000 dilution of either rabbit polyclonal antibody against LC3 or rabbit monoclonal antibody against Beclin 1 (Cell Signaling Technology, Danvers, MA, USA), or a horseradish peroxidase (HRP)-conjugated β-actin monoclonal antibody (1:20,000 dilution; Sigma-Aldrich). The membranes were then incubated with a HRP-conjugated anti-rabbit immunoglobulin G (1:6,000 dilution; Sigma-Aldrich) secondary antibody, for 1 h. The membranes were washed three times with PBS-Tween^®^, between each antibody incubation. The protein bands were visualized using an Enhanced Chemiluminescence Western Blot Analysis system (Pierce Biotechnology, Inc., Rockford, IL, USA), and quantified by densitometry using Quantity One Image Analysis Software (Bio-Rad Laboratories).

### siRNA transfection

siRNA sequences specifically targeting human Beclin 1, and non-target control sequences were constructed by Genepharma Co., Ltd. (Shanghai, China). The sequences used were as follows: Beclin 1 siRNA sense, 5′-CGGCUCCUAUUCCAUCAAATT-3′, and anti-sense, 5′-UUUGAUGGAAUAGGAGCCGTT-3′; control siRNA sense, 5′-UUCUCCGAACGUGUCACGUTT-3′, and anti-sense, 5′-ACGUGACACGUUCGGAGAATT-3′. A total of 2×10^5^ cells/well were seeded into 6-well plates, and the following day were transfected with 100 nM final concentration Beclin 1 siRNA, using Lipofectamine^®^ 2000 reagent (Invitrogen Life Technologies, Carlsbad, CA, USA), according to the manufacturer’s instructions. The cells were collected 48 h following transfection, for cell viability and apoptosis assays.

### Immunofluorescence

The A2780 and A2780cp cells were grown on round glass coverslips (Fisher Scientific, Waltham, MA, USA) in 35 mm cell culture dishes. Following a 20 min fixation with pre-chilled methanol, the coverslips were washed with PBS, permeabilized with 0.2% Triton X-100-PBS for 15 min, and blocked with 2% bovine serum albumin-PBS for 30 min. The coverlips were then incubated with rabbit polycolonal p62 and goat polyclonal LC3 primary antibodies (1:50) at 37°C for 90 min in the dark, followed by three 10 min washes in PBS. Subsequently, the coverlips were incubated with goat-anti rabbit IgG-TR and donkey anti-goat IgG-FITC secondary antibodies, respectively (1:1,000) at 37°C for 1 h in the dark, and washed three times (10 min/wash) with PBS. All of the antibodies were purchased from Santa Cruz Biotechnology (Dallas, TX, USA). The coverslips were mounted onto glass slides, with antifade mounting medium purchased from Invitrogen (Paisley, UK). The images were captured using a Zeiss Observer.Z1 microscope (Zeiss, Oberchoken, Germany) and Slidebook 4.2.0.11 computer software (Intelligent Imaging Innovations, Inc., Denver, CO, USA).

### Transmission electron microscopy

The cells were fixed using 2.5% glutaraldehyde in 0.1 M phosphate buffer for 2 h at 4°C, and then post-fixed using 1% osmium tetroxide for 3 h. The samples were scraped and pelleted, dehydrated in a graded series of ethanol baths, infiltrated, and embedded in Epon™ resin. Ultrathin sections (70 nM) were cut using a Leica Ultracut Microtome (Leica Microsystems Inc., Buffalo Groce, Il, USA), stained with uranyl acetate for 3 min, and examined using a JEOL JEM-1400 transmission electron microscope (JEOL Ltd., Tokyo, Japan).

### Apoptosis analysis

For the assessment of the cellular apoptotic rate, the fluorescein isothiocyanate Annexin V Apoptosis Detection kit I (BD Pharmingen, San Diego, CA, USA) was used. Following 48 h of treatment, the cells were collected and centrifuged at 3,190 × g for 5 min. The cells were then resuspended in 500 μl binding buffer and stained with 5 μl Annexin V and 5 μl propidium iodide (PI), for 15 min at room temperature in the dark. The samples were analyzed by flow cytometric analysis (FC500 MPL, Beckman Coulter, Brea, CA, USA). A total of 2,000 events were measured and the results are presented as the percentage (Annexin V positive) of apoptotic cells.

### Statistical analysis

The data are presented as the means ± standard deviation. A two-tailed student’s t-test was used to compare the differences between two groups. The analyses were performed using SPSS version 16.0 (SPSS, Inc., Chicago, IL, USA) software. P<0.05 was considered to indicate a statistically significant difference.

## Results

### A2780cp cells are resistant to cisplatin-induced cell death

**Figure 1:**
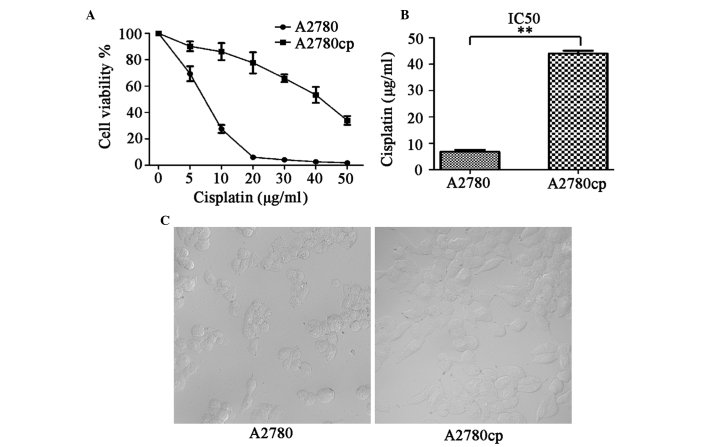
Effects of cisplatin in A2780 and A2780cp ovarian cancer cell lines. (A) The cells were treated with different concentrations of cisplatin (0–50 μg/ml) for 24 h. The cell viability was determined using a water soluble tetrazolium salt-8 assay. The data are represented as the means ± standard deviation, of three independent experiments; ^*^P<0.05. (B) The half maximal inhibitory concentration (IC_50_) values of cisplatin in both cell lines were calculated. Each value represents the means ± standard deviation, of three independent experients; ^**^P<0.01. (C) The cell morphology of both cell lines as observed using phase-contrast microscopy (magnification, 400x).

To verify that the A2780cp cells were resistant to cisplatin, the parental A2780 and A2780cp cells were treated with increasing concentrations of cisplatin (5–50 μg/ml) for 24 h, and the cell viability was measured using a WST-8 assay. The percentage of surviving cells decreased in a dose-dependent manner in both the A2780 and A2780cp cells (Fig. 1A). However, as expected, the A2780cp cells were 6.5× more resistant to cisplatin, as compared with the A2780 parental cells (P<0.01). The 24 h half maximal inhibitory concentrations (IC_50_) of cisplatin in A2780cp and A2780 cells, were 44.07±1.1 and 6.84±0.66 μg/ml, respectively (Fig. 1B). Using phase-contrast microscopy, the A2780cp cells were observed as having a regular, round shape, and were markedly larger as compared with the A2780 cells (Fig. 1C).

### Autophagy is involved in cisplatin resistance in ovarian cancer cells

**Figure 2:**
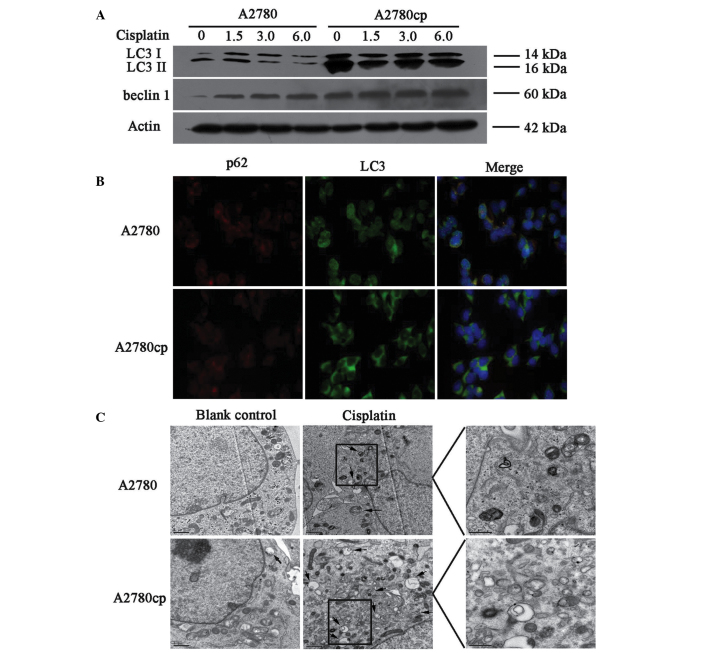
Autophagy induced by cisplatin treatment in A2780 and A2780cp ovarian cancer cells. (A) The cells were treated with different concentrations of cisplatin (0, 1.5, 3, 6 μg/ml) for 24 h. The cell lysates were collected for western blot analysis using antibodies against microtubule-associated protein 1 light chain 3 (LC3) and Beclin 1. (B) Indirect immunofluorescence of LC3 and p62 was performed in both cell lines, the red signal represents the p62 levels, and the green signal represents the LC3 levels (magnification, 40x). (C) Representative electron microscopy images of autophagosomes (magnification, 10,000x). The scale bars represent 1 μm, and the arrows indicate the autophagosomes. kDa, kilodaltons.

The endogenous levels of autophagy were compared in the A2780cp cisplatin-resistant and A2780 sensitive cell lines, by measuring the protein expression levels of LC3 II and Beclin 1. A2780cp cells exhibited higher expression levels of LC3 II and Beclin 1 proteins, as compared with the A2780 cells (Fig. 2A, lanes 1 and 5). Immunofluorescence staining for LC3 and p62, another autophagy-related protein, also showed that the cisplatin-resistant cells exhibited higher levels of autophagy (Fig. 2B). These findings suggest that increased levels of autophagy may contribute to cisplatin resistance in ovarian cancer cells.

The present study also determined whether cisplatin treatment induced autophagy in both of the cell lines. The cells were treated with 1.5, 3 and 6 μg/ml cisplatin for 24 h, and then the protein expression levels of LC3 and Beclin 1 were determined. As shown in Fig. 2A, cisplatin induced the protein expression of LC3 II and Beclin 1 in both cell lines. Notably, the protein expression levels of Beclin 1 in both cell lines was increased in a dose-dependent manner, whereas LC3 II did not change (Fig. 2B and C). Electron microscopic analyses also revealed that treatment with cisplatin increased the amount of autophagosomes in both cell lines; however, the number of autophagosomes increased to a greater extent in the A2780cp cells, as compared with the A2780 cells (Fig. 2C). These findings suggest that cisplatin may induce autophagy in ovarian cancer cell lines, and the induced level of autophagy was higher in A2780cp cells, as compared with that in A2780 cells.

### Inhibition of autophagy sensitizes cells to cisplatin treatment

**Figure 3:**
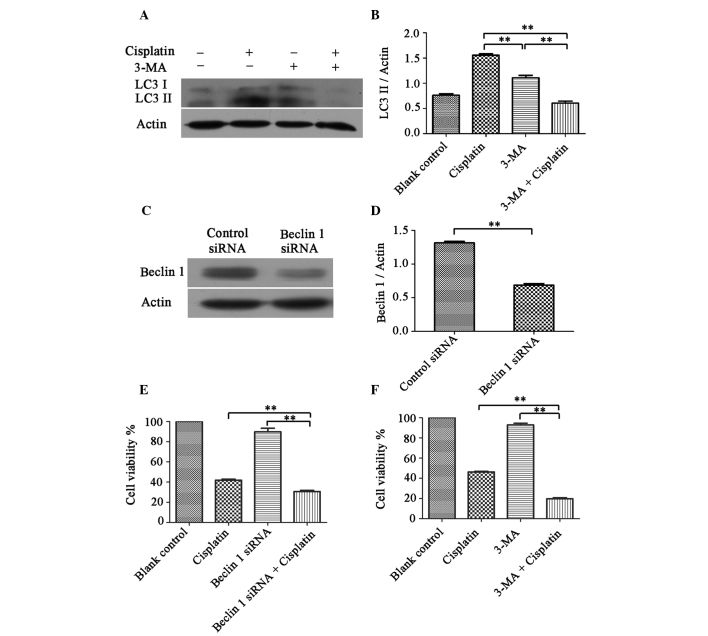
Effects of 3-methyladenine (3-MA) and Beclin 1 small interfering (si)RNA on the cell viability of A2780cp ovarian cancer cisplatin-resistant cells. (A) The cells were treated with or without cisplatin (6 μg/ml) and 3-MA (1 mmol), for 24 h. The cell lysates were collected for western blot analysis using antibodies against microtubule-associated protein 1 light chain 3 (LC3) I and II. (B) The relative LC3 II protein expression levels were normalized to β-actin (C) The cells were transiently transfected with control or Beclin 1 siRNA (100 nM) for 48 h. The cell lysates were collected for western blot analysis using an antibody against Beclin 1. (D) The relative Beclin 1 protein expression levels were normalized to β-actin. (E) Following 48 h transfection, the cells were treated with cisplatin for 24 h and the cell viability was determined using a WST-8 assay. (F) The cell viability, following 3-MA treatment (+/−), was determined using a water soluble tetrazolium salt-8 (WST-8) assay; ^**^P<0.01. The data represent the means ± standard deviation, of three experiments.

A2780cp cells were shown to have elevated levels of autophagy. Therefore, to determine whether they could be re-sensitized to cisplatin, the cells were treated with an inhibitor of autophagy, 3-MA, or a Beclin 1 targeting siRNA. A2780cp cells were pretreated with 1 mmol 3-MA for 1 h, followed by cisplatin (6 μg/ml) for 24 h. The cell lysates were subjected to western blot analysis, to determine the protein expression levels of LC3 I and LC3 II. As shown in Fig. 3A and B, treatment with 3-MA decreased the LC3 II protein expression levels induced by cisplatin and increased cisplatin-induced cell death in A2780cp cells (Fig. 3F). As shown in Figure 3C and D, the Beclin 1 siRNA treatment group had a ~50% reduction in Beclin 1 expression, as compared with the control siRNA treatment group. Furthermore, knockdown of Beclin 1 expression sensitized A2780cp cells to cisplatin, by enhancing cisplatin-induced cell death (Fig. 3E).

### Inhibition of autophagy enhances cisplatin-induced apoptosis

**Figure 4:**
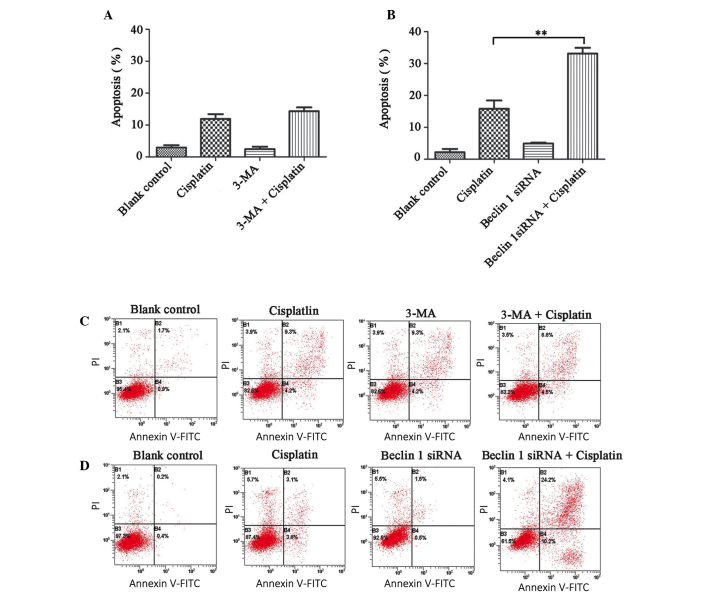
Effects of 3-methyladenine (3-MA) and Beclin 1 small interfering (si)RNA on the apoptosis of A2780cp ovarian cancer cisplatin-resistant cells. (A) The apoptotic rate of cells treated in the absence, or presence, of cisplatin and 3-MA was determined. (B) The apoptotic rate of cells treated in the absence or presence of cisplatin and Beclin 1 siRNA transfection was determined. The data are represented as the means ± standard deviation, of three independent experiments. ^**^P< 0.01 (C) The cells were treated with or without cisplatin (6 μg/ml) and 3-MA (1 mmol) for 24 h. (D) The cells were transfected with Beclin 1 siRNA (100 nM) for 48 h, and treated with or without cisplatin for 24 h. All of the apoptotic levels were determined using the fluorescien isothiocyanate (FITC) Annexin V apoptosis assay. PI, propidium iodide.

To determine whether the inhibition of autophagy influenced cisplatin-induced apoptosis in A2780cp cells, an apoptosis assay was performed following a co-treatment of cisplatin, with either 3-MA or Beclin 1 siRNA transfection. The apoptotic rate (at both the early and advanced stages) of the control, 3-MA and Beclin 1 siRNA groups were 2.96±0.72, 2.43±0.79 and 4.92±0.28%, respectively (Fig. 4A and B). Furthermore, the 3-MA plus cisplatin group did not increase the apoptotic rate of the cells, as compared with the cisplatin only group (14.35±1.78 vs. 11.91±1.49%, P>0.05; Fig. 4A and C). However, the Beclin 1 siRNA plus cisplatin group, had an increased percentage of apoptotic cells, as compared with the cisplatin only group (33.14±1.78 vs. 15.79±2.62%, P<0.05, Fig. 4B and D).

## Discussion

Cisplatin resistance is a major obstacle in the successful treatment of cancer, including ovarian cancer. Previous studies have attempted to elucidate the mechanisms responsible for cisplatin resistance in cancer. A prominent hypothesis suggests that resistant cells fail to undergo apoptosis following cisplatin treatment. Consequently, numerous studies have attempted to use anti-apoptotic inhibitors, to sensitize resistant cancer cells and tumors to chemotherapeutics. However, these studies have demonstrated that targeting apoptosis does not optimally inhibit chemoresistance (13,14). Therefore, alternative cell-death pathways, such as autophagy, have become an important area of research; however, the role of autophagy in cancer chemoresistance remains unclear. Previously, a study using SKOV3 ovarian cancer cells, revealed that cisplatin-resistant ovarian cancer cells expressed high levels of autophagy. However, this study focused on the ubiquitin protein p62, which was shown to regulate autophagy degradation, prevent endoplasmic reticulum stress-induced apoptosis and lead to cisplatin resistance in human ovarian cancer cells, but provided limited information on the role of autophagy in cisplatin resistance (15).

To further explore the role of autophagy in cisplatin resistance, the present study used A2780 and A2780cp cells as a model for *in vitro* analysis. A cell viability assay confirmed that A2780 and A2780cp cells provide an ideal pair of cell lines to use for these studies, since A2780cp cells were 6.5× more resistant to cisplatin, as compared with the parental cell line. The level of autophagy was evaluated in both the ovarian cancer cell lines. Autophagy is regulated through a family of ATG genes. Beclin 1, a mammalian autophagy gene, is generally combined with class III phosphoinositide 3-kinase, as a complex which has been shown to be necessary for the initiation of autophagy (16). During the formation of an autophagosome, LC3 is cleaved to produce its active form: LC3 I, which conjugates with phosphatidylethanolamine to form LC3 II, which is localized to the autophagosomal membrane (17). Therefore, LC3 II may be examined as an indicator of autophagy activity. P62 is a polyubiquitin-binding protein, which binds directly to LC3 and is degraded by autophagy activation (18,19). In the present study, following the treatment of the cells with different concentrations of cisplatin for 24 h, both autophagy markers, LC3 and Beclin 1 were upregulated in A2780cp cells, as compared with the A2780 cells, as determined by western blot analysis. Furthermore, untreated A2780cp cells expressed greater amounts of LC3 and a lower amount of p62, as determined by immunofluorescence, as compared with the A2780 cells. These results suggested that autophagy was more active in cisplatin-resistant cells. The previous SKOV3 ovarian cancer cell study examined the accumulation of LC3 by western blot analysis, to distinguish autophagy levels in the cells (15). To confirm that chemoresistant ovarian cancer cells expressed higher levels of autophagy, LC3 and Beclin 1 protein expression levels were evaluated by western blotting, alongside the amount of p62 and LC3 through indirect immunofluorescence, and the number of autophagosomes by transmission electron microscopy. The levels of autophagy increased in response to cisplatin, in a dose dependent manner, in the A2780cp resistant cells. These results suggested that there is a protective role of autophagy in cisplatin resistance.

To explore whether inhibiting autophagy may sensitize resistant cells to cisplatin treatment, the effects of an inhibitor of autophagy or Beclin 1 siRNA were examined on cell death, in A2780cp cells. 3-MA is a specific inhibitor of the autophagic pathway, which functions by inhibiting the class III phosphatidylinositol 3-kinases and blocking the formation of autophagosomes at the sequestration step (20,21). Previously, in EC9706 esophageal squamous carcinoma cells, 3-MA was shown to contribute to the upregulation of cisplatin-induced cell death (22). In the present study, low doses of 3-MA (1 mmol) suppressed LC3 II protein formation and sensitized A2780cp cells to cisplatin treatment. Beclin 1 has a critical, regulatory role in autophagy, and downregulation of Beclin 1 has previously been shown to sensitize Hela human cervical cancer cells and HepS mouse liver cancer cells to cisplatin (23). In the present study, knockdown of Beclin 1 expression, using siRNA, significantly inhibited autophagy and sensitized A2780cp cells to cisplatin treatment. Furthermore, the apoptotic rate of the cells was analyzed, to explore the potential mechanisms of autophagy inhibition on cisplatin sensitization. Previously, in HT29 human colorectal cancer cells, 3-MA treatment enhanced 5-FU-induced apoptosis (24). In the present study, however, the apoptotic rates were not markedly altered following a co-treatment of cisplatin with 3-MA. Similar effects were observed in esophageal cancer cells, in which disruption of lysosomal activity with the pharmacological inhibitors bafilomycin A1 or chloroquine did not improve the chemotherapeutic effects (9). A possible explanation is that pharmacological inhibitors exert transient effects, which may result in the activation of another potential cell-death mechanism, induced by cisplatin. However, knockdown of Beclin 1 expression, with siRNA, significantly inhibited autophagy and increased cisplatin-induced apoptosis. This finding is consistent with a previous study, which indicated that the cleavage of Beclin 1 reduced autophagy and promoted apoptosis in HeLa cells (25). These findings suggest that Beclin 1 may be a potential target for autophagy inhibition, in order to sensitize cancer cells to chemotherapy.

In conclusion, higher levels of autophagy were observed in cisplatin-resistant ovarian cancer cells, whereas knockdown of Beclin 1 expression restored cisplatin-sensitivity to these cells, which is attributed to autophagy inhibition. Therefore, targeting autophagy may be a potential therapeutic strategy in ovarian cancer treatment.
